# Bone adaptation to mechanical loading in a mouse model of reduced peripheral sensory nerve function

**DOI:** 10.1371/journal.pone.0187354

**Published:** 2017-10-31

**Authors:** Mollie A. Heffner, Damian C. Genetos, Blaine A. Christiansen

**Affiliations:** 1 University of California Davis, Mouse Biology Program, Davis, CA, United States of America; 2 University of California Davis School of Veterinary Medicine, Department of Anatomy, Physiology & Cell Biology, Davis, CA, United States of America; 3 University of California Davis Medical Center, Department of Orthopaedic Surgery, Sacramento, CA, United States of America; University of Zaragoza, SPAIN

## Abstract

Underlying mechanisms contributing to the imbalance in bone turnover during osteoporosis remain only partially explained. Reduced sensory nerve function may contribute to this imbalance, as sensory neuropeptides affect the activity of osteoblasts and osteoclasts *in vivo*, especially during bone adaptation. In this study, we investigated bone adaptation in mice following two weeks of tibial compression (peak magnitude 3 N or 7 N). To induce decreased sensory nerve function, mice were treated with capsaicin as neonates. We hypothesized that decreased sensory nerve function would diminish the adaptation of bone to mechanical loading, assessed with μCT and dynamic histomorphometry. We found that tibial compression induced significant changes in cortical microarchitecture that depended on compression magnitude and location along the length of the tibia; in contrast, there was no effect of loading on trabecular bone of the tibial metaphysis. Tibial compression significantly increased periosteal, and decreased endosteal, bone formation. Contrary to our initial hypothesis, capsaicin-treated mice generally displayed a similar, if not larger, adaptive response to mechanical loading, including greater increases in bone mineral content and mineral apposition rate. To integrate mechanical loading of bone with sensory nerve activation, we examined whether concentration of the neuropeptides calcitonin gene-related peptide (CGRP) and substance P (SP) in bone were affected following 1 or 5 days of 5 N tibial compression or hindlimb unloading. We found that 1 day of tibial compression significantly increased CGRP concentrations in bone, and hindlimb unloading also exhibited a trend toward increased CGRP in bone. These results may suggest a role of sensory nerves in the bone adaptation response to the mechanical environment, though this remains unclear.

## Introduction

Over half of Americans 50 years and older have osteoporosis or low bone mass [1]. With age, there is an imbalance in bone turnover, with bone resorption exceeding formation, resulting in a net loss of bone. The underlying causes of this imbalance in bone remodeling are not fully understood. Innervation of bone provides a potential mechanism for influencing bone metabolism. Bone micrographs reveal a dense neural network [2, 3] suggesting a role for nerves in skeletal development or adaptation independent of sensation. *In vitro*, osteoblasts and osteoclasts express neuropeptide receptors, the activation of which influence bone cell differentiation and function [4–7]. Furthermore, studies of genetic mouse models have shown that alterations to nerves can affect bone density [8, 9]. Together, these studies demonstrate that sensory nerves could influence bone metabolism through neuropeptide action on bone cells.

Calcitonin gene related peptide (CGRP) and substance P (SP) are two neuropeptides found in the sensory nerves innervating bone. Osteoblasts respond to CGRP with an increase in intracellular cAMP and corresponding changes in cell morphology and function [7]. CGRP also affects osteoclasts, inhibiting their function and the recruitment of macrophages into osteoclast-like cells [10–12]. *In vivo* administration of CGRP in rats and rabbits inhibited bone resorption and lowered serum concentrations of calcium [13, 14]. Osteoblasts and osteoclasts also express SP receptors, and exposure to SP increases osteoblast function *in vitro* [15], while exposure of osteoclasts to SP stimulates calcium influx and bone resorption [16]. CGRP may facilitate SP release and enhance activation [17]. *In vivo*, increased mechanical loading (cyclic ulnar compression) of rat ulnae decreased bone CGRP and SP concentrations from one hour to 10 days after a single round of loading [18]. In contrast, both CGRP and SP levels were increased in the sciatic nerve after 4 weeks of decreased mechanical loading (cast immobilization) in rats [19]. These findings provide evidence for a role of neuropeptides in the regulation of local bone cell function in the context of mechanical loading or unloading.

The interaction between sensory nerves and bone can be studied using capsaicin, a natural irritant compound. Treatment of neonatal mice with capsaicin destroys unmyelinated and small diameter myelinated sensory neurons [20, 21]. This mouse model has been extensively used for studies of itch [22, 23] and pain [24], and may also be useful for investigating the role of peripheral sensory nerves in bone metabolism and bone adaptation. A previous study using capsaicin treatment in adult rats found that capsaicin treatment induced a loss of trabecular bone, increase in osteoclast number, decrease in osteoblast activity, and 60–70% reduction in SP and CGRP concentrations in bone tissue 4 weeks after treatment [25]. However, since these rats were treated with capsaicin as adults, it is difficult to determine if these bone changes were due to decreased sensory nerve activity, or were a direct consequence of the capsaicin treatment itself. We previously examined the role of peripheral sensory nerves in skeletal development by treating neonatal mice with capsaicin; these mice demonstrated prolonged reductions in thermal sensitivity through 12 weeks of age, and modest reductions in trabecular thickness at the distal femoral metaphysis compared to vehicle-treated mice [26]. The small changes observed in bone during development suggest that capsaicin-sensitive neurons may not have a considerable role in bone metabolism under normal loading conditions. However, it is possible that decreased peripheral sensory nerve function may be important during bone adaptation. In a study with capsaicin-treated rats, Hill *et al*. found that normal tibial growth was not affected by capsaicin treatment, but osteoclast recruitment induced by maxillary molar removal was significantly lower in capsaicin-treated rats compared to control rats [27]. Similarly, Sample *et al*. reported that perineural anesthesia of the brachial plexus diminished the adaptive response of bone to ulnar compression [18].

In order to investigate the role of sensory nerves in bone adaptation, we subjected mice treated with capsaicin as neonates to two weeks of tibial compression. Capsaicin- and vehicle-treated mice received cyclic tibial compression at magnitudes of either 3 N or 7 N, and adaptation was quantified using μCT and dynamic histomorphometry. We hypothesized that the reduced sensory nerve function in capsaicin-treated mice would impair bone adaptation to mechanical loading. Additionally, to investigate how neuropeptide concentrations were altered in response to mechanical loading or unloading, we subjected mice to 1 or 5 days of increased mechanical loading (5 N tibial compression) or decreased mechanical loading (hindlimb unloading), and CGRP and SP levels were measured in the tibia. We hypothesized that concentrations of both neuropeptides would be decreased by tibial compression and increased by hindlimb unloading.

## Materials and methods

### Animals

**Fig 1:**
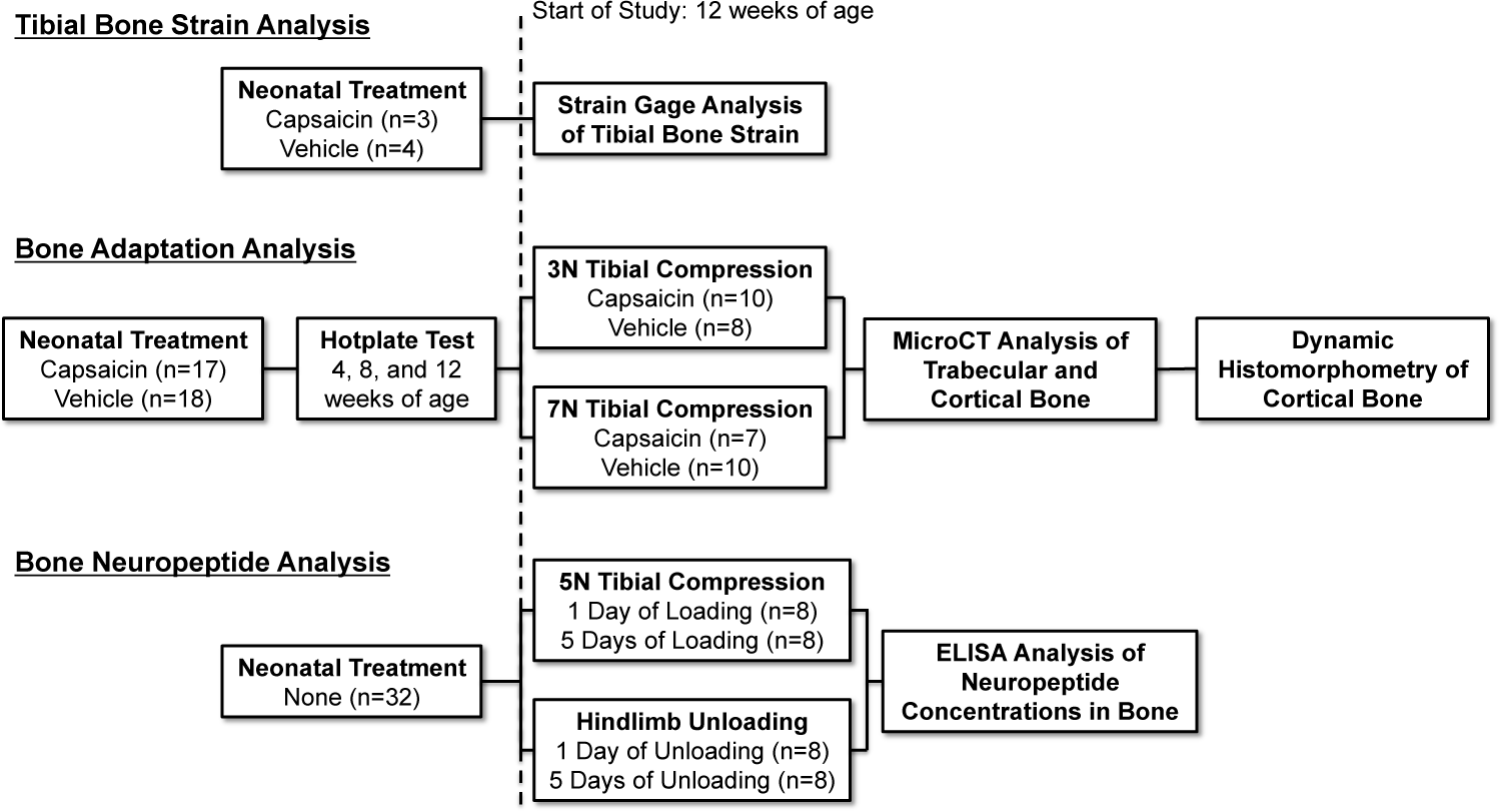
Schematic of experimental design.

A total of 74 female C57BL/6 mice were used for these studies (Fig 1; Harlan Laboratories, Indianapolis, IN). 35 mice were treated with either capsaicin or vehicle as neonates; these mice were randomized into groups based on treatment and tibial compression loading magnitude (n = 7–10 per treatment/magnitude). 32 mice had no treatment, and were subjected to tibial compression or hindlimb unloading in order to quantify changes in neuropeptide concentrations in bone (n = 8 per stimulus/time point). Seven mice were treated with either capsaicin (n = 3) or vehicle (n = 4) as neonates; these mice were used to determine bone strain magnitudes during tibial compression. Mice were cared for in accordance with the guidelines set by the National Institutes of Health (NIH) on the care and use of laboratory animals. Mice were housed in Tecniplast conventional cages (Tecniplast SPA, Buguggiate, Italy), with Bed-o’ Cob bedding (The Andersons Inc., Maumee, OH), with 4 mice per cage, 12 hour light/dark cycle, 20–26°C ambient temperature. Mice had ad libitum access to food (Harlan irradiated 2918 chow) and autoclaved water, and were provided with Enviro-dri and/or Nestlets for environmental enrichment. Mice were monitored by husbandry staff at least once a day, 7 days a week, with monthly health care checks by a veterinarian. All mice were euthanized by CO2 asphyxiation, immediately followed by cervical dislocation. All procedures were approved by the institutional Animal Studies Committee at UC Davis.

### Neonatal capsaicin treatment

Neonatal capsaicin treatment was performed as previously described [28]. Briefly, neonatal mice were given subcutaneous injections of capsaicin (50 mg/kg) or vehicle (10% ethanol, 10% Tween 80 in isotonic saline) on day 2 and 5 after birth (n = 18 vehicle, 17 capsaicin). Previous studies have shown that this treatment protocol decreases peripheral sensory nerve function for the lifetime of the animal, but motor function is not affected [29]. Following capsaicin or vehicle treatment, neonatal mice were returned to normal cage activity until weaning (28 days). Mice were subjected to tibial compression starting at 12 weeks of age with magnitudes of either 3 N or 7 N, with a total of 7–10 mice/treatment group/compressive force.

### Tibial compression loading

The tibial compression loading protocol was similar to that used by others in studies of bone adaptation [30, 31]. The tibial compression system consisted of two custom-built loading platens, positioned vertically within an electromagnetic materials testing machine (ElectroForce 3200, TA Instruments, New Castle, DE). The bottom platen held the knee flexed to approximately 90°, and the top platen positioned the foot with the ankle flexed to approximately 30°. A slight preload (<0.5N) maintained the position of the limb. Mice were anesthetized with isoflurane inhalation while the right lower leg of each mouse was subjected to cyclic axial compressive loading for 1200 cycles per day. Capsaicin and vehicle-treated mice were loaded for a total of 10 days (M-F) over two weeks. For 18 of the mice, the top platen was driven at 5.5 mm/sec to a target compressive load of 3 N. The remaining 17 mice were loaded at 8.5 mm/sec to a target compressive load of 7 N. The 3 N load magnitude was selected because it is the lowest load magnitude previously shown to induce an anabolic response in bone [30]. The 7 N load magnitude was chosen as a load that would cause a considerable bone formation response, but would avoid anterior cruciate ligament (ACL) injury, which we have previously noted at 9–10 N tibial compression in mice [32]. Mice were euthanized the third day after completion of two weeks of tibial compression. Mice used for quantification of neuropeptides (n = 16) were subjected to cyclic axial compressive loading for a total of 1 day (mice were sacrificed within an hour of the second bout of loading) or 5 days (mice were sacrificed within an hour of the fifth bout of loading). For these mice, the top platen was driven at 5.5 mm/sec to a target compressive load of 5 N. The 5 N load magnitude was selected as the midpoint between the “low” and “high” magnitude loading protocols used for quantification of bone adaptation. A saw-tooth loading protocol was used, with a 0.1 s dwell between each load cycle at the pre-load level of 0.5 N. All loading rates resulted in a load frequency of approximately 4 cycles/second. Left tibias were used as internal controls. Mice were weighed on days 1, 5 and 10 of loading.

### Hindlimb unloading

Mice subjected to hindlimb unloading for quantification of neuropeptides (n = 16) were tail-suspended as previously described [33]. Mice were housed individually and suspended at a head-down angle of approximately 30°so that the hindlimbs were not able to touch the cage floor. The tail was secured to a metal apparatus hung from a swivel and pulley above the cage floor, permitting movement throughout the cage and rotation of 360°. Metal grid flooring was used to facilitate mouse movement. Mice were provided with Enviro-dri and/or Nestlets for environmental enrichment. Water and food were available throughout the experimental period. Mice were monitored daily for signs of distress resulting from the tail suspension protocol. Mice were suspended for either 1 day (24 hours) or 5 days (120 hours).

### Tibial bone strain measurement

A preliminary study was performed to quantify tibial strain in 12 week-old capsaicin- (n = 3) and vehicle-treated mice (n = 4) during axial compression. Immediately after sacrifice, a single element strain gauge (UFLK-1-11-1L, Tokyo Sokki Kenkyujo Co., Ltd., Japan) was bonded to the anteromedial surface of the tibia in alignment with the long axis of the bone. The center of the gauge was located 5 mm distal to the tibial plateau. This location is commonly used for measuring bone strain during tibial compression in mice [31, 34, 35] since positioning of the strain gauge is limited by tibia size and morphology. The strain gauge was wired to amplification circuitry and the data acquired in LabView (National Instruments, Santa Clara, CA). The lower leg of each mouse was compressed in intervals of 1 N from 1 N to 7 N at a loading rate of 5.5 mm/sec.

### Hot-plate analgesia testing

Capsaicin- and vehicle-treated mice were subjected to hot-plate analgesia testing at 4, 8, and 12 weeks of age (before the start of tibial compression) to determine response time to a constant thermal stimulus of 55°C as previously described [36]. Mice were placed on a hot-plate (LE 7406, Coulborn Instruments, Whitehall, PA) and removed after indication of discomfort, determined as twitching or licking of a hind limb or jumping, or after a maximum of 30 seconds, and the latency time of the response was recorded. Each mouse was tested twice at each age, with approximately 2 hours between tests, and the latency times were averaged for each mouse.

### Micro-computed tomography analysis of bone structure

**Fig 2:**
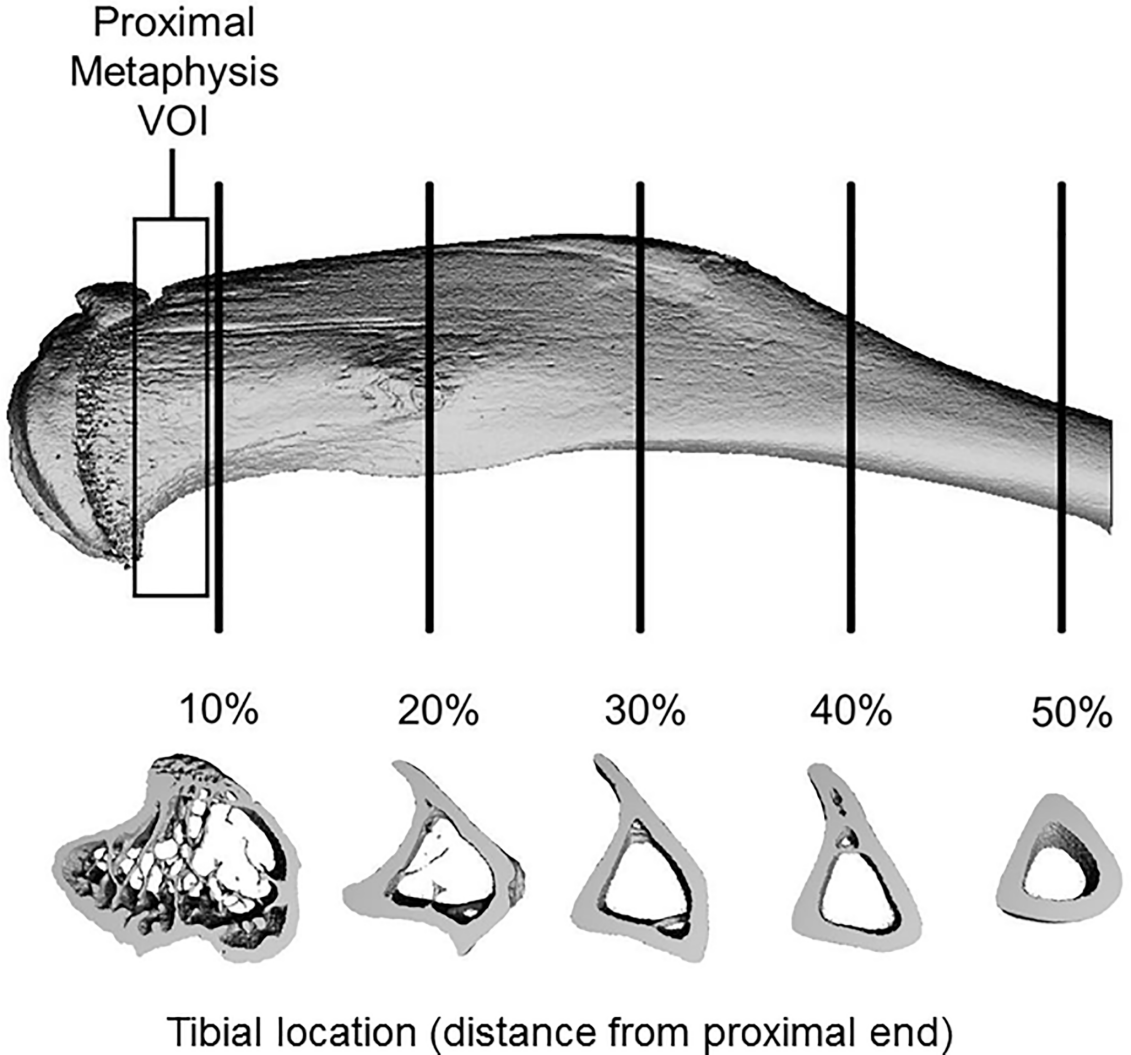
Regions of interest for analysis of trabecular and cortical bone. MicroCT analysis of trabecular bone was performed at the proximal tibial metaphysis. Cortical bone analysis was performed using μCT and dynamic histomorphometry at 5 locations along the length of the tibias.

Bilateral tibias of capsaicin- and vehicle-treated mice were removed post mortem and preserved in 70% ethanol. Bones were scanned using micro-computed tomography (SCANCO, μCT 35, Brüttisellen, Switzerland); images were acquired at 6 μm nominal voxel size (x-ray tube potential = 55 kVp, intensity = 114 μA, integration time = 900 ms). Trabecular bone was analyzed at the metaphysis of the proximal tibia using manually drawn contours inside the cortical shell on two-dimensional slices. The metaphysis was defined by a 900 μm thick volume of interest beginning below the middle break of the growth plate. Trabecular bone volume per total volume (BV/TV), trabecular thickness (Tb.Th), trabecular number (Tb.N), tissue mineral density (TMD) and other structural outcomes were determined using the manufacturer’s 3-D analysis tools. Cortical bone was analyzed at 10, 20, 30, 40 and 50% of the tibia lengths (a region encompassing peak compressive strain [37] and known cortical bone response [38]; Fig 2), using 600 μm thick volumes of interest centered at the measured locations. Total cross-sectional area (Tt.Ar), cortical bone area (Ct.Ar), medullary area (Me.Ar), cortical thickness (Ct.Th), and bone mineral content (BMC) were determined using the manufacturer’s 3-D analysis tools.

### Dynamic histomorphometry of bone formation

Capsaicin- and vehicle-treated mice received injections of calcein green (10 mg/kg; Sigma-Aldrich, St. Louis, MO) and Alizarin-3-methlimino-diacetic acid (30 mg/kg; Sigma-Aldrich, St. Louis, MO) 10 days and 3 days prior to sacrifice, respectively. After scanning with μCT, tibias were embedded in Technovit (Kulzer, Wehrheim, Germany) using standard techniques for undecalcified bone [39]. Sections were cut from each bone on a bandsaw (Model 310, Exakt Technologies, Norderstedt, Germany) in the transverse plane at 10, 20, 30, 40 and 50% of the tibia length (Fig 2). Sections were ground to an approximate thickness of 100 μm. Fluorescent images were obtained at 10x magnification (Nikon Eclipse TE2000-E, Tokyo, Japan). Dynamic histomorphometric analysis was performed using commercial software (Bioquant, Nashville, TN). Mineral apposition rate (MAR), percent mineralizing surface (MS/BS), and bone formation rate (BFR/BS) were quantified for the endosteal and periosteal surfaces of cortical bone at each location.

### ELISA analysis of neuropeptide concentrations in bone

Tibias were collected from mice immediately after euthanasia for quantification of whole bone concentrations of CGRP and SP. Tibias were flash frozen in liquid nitrogen, and transferred to a -80°C freezer until protein isolation. Frozen whole bones were cut in half and homogenized in Trizol for isolation. Protein was analyzed in duplicate to determine the concentrations of CGRP and SP using commercial mouse-specific ELISAs (Cusabio, Wuhan, China) per the manufacturer’s instructions. Data were normalized to total protein concentration to account for variation in extraction efficiency.

### Statistical analysis

Hot-plate data were analyzed using 2-way ANOVA stratified by compressive force and treatment. Between-group differences were analyzed using a Tukey’s HSD post hoc test. Body weight data were analyzed using a repeated measures approach with factors for stimulus day, type of stimulus, and the interaction. Between-group differences were analyzed using Student’s t-test. To account for repeated measures and paired data, μCT and histomorphometry data were analyzed separately using mixed effects models with factors for treatment (capsaicin or vehicle), leg (contralateral or loaded), volume of interest (10–50% of tibia length) and interactions. Between-group differences were analyzed using Tukey’s HSD post hoc test when the factor was significant and the model had an adjusted R squared greater than 0.9. ELISA data for each neuropeptide were analyzed using two-way ANOVA stratified by stimulus type, stimulus duration, and the interaction. Between-group differences were analyzed using Tukey’s HSD post hoc test. All data were analyzed using the Shapiro-Wilk test of normality. All data were found to be normally distributed except for endocortical MAR, periosteal MAR, and some neuropeptide ELISA data. These data were further analyzed using the Kruskal-Wallis test by ranks. All data were analyzed using JMP (SAS Institute Inc., Cary, NC). Data are reported as mean ± SD. Significance was defined as p<0.05.

## Results

### Animal body mass

**Fig 3:**
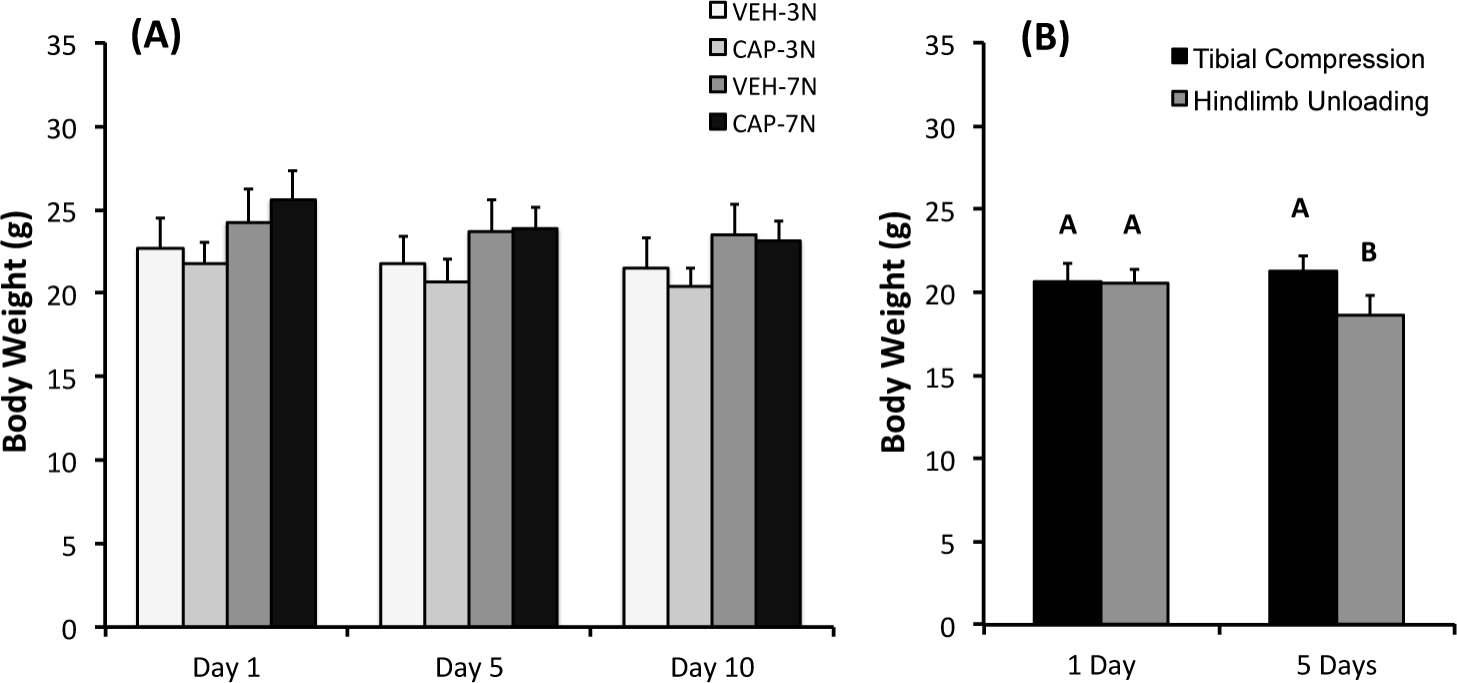
Body weights of mice during tibial compression or hindlimb unloading. (**A**) Body weights of capsaicin- and vehicle-treated mice subjected to tibial compression at 3 N or 7 N. (**B**) Body weights of mice subjected to 5 N tibial compression or hindlimb unloading. Groups not connected by the same letter are significantly different (*p* < 0.05).

Tibial compression was generally well tolerated by capsaicin- and vehicle-treated animals, as mice returned to normal cage activity after waking from anesthesia, and did not exhibit obvious signs of pain or distress. Body weights of capsaicin- and vehicle-treated mice were not different on loading day 1, and changes in body weight were similar between treatment groups (Fig 3A). Average weight loss between loading days 1 and 10 was 5.6% of body weight for all experimental groups. For mice used to quantify neuropeptides in bone, there was a significant effect of time point (p = 0.03), type of stimulus (tibial compression, hindlimb unloading, or control; p = 0.009), and the interaction (p = 0.0003) on body weight. There was not a difference in body weight between groups on loading day 1, but on day 5 the hindlimb unloaded mice exhibited a significant decrease in body weight (Fig 3B). Hindlimb unloaded mice lost an average of 9% body weight during 5 days of tail suspension.

### Tibial bone strain measurement

**Fig 4:**
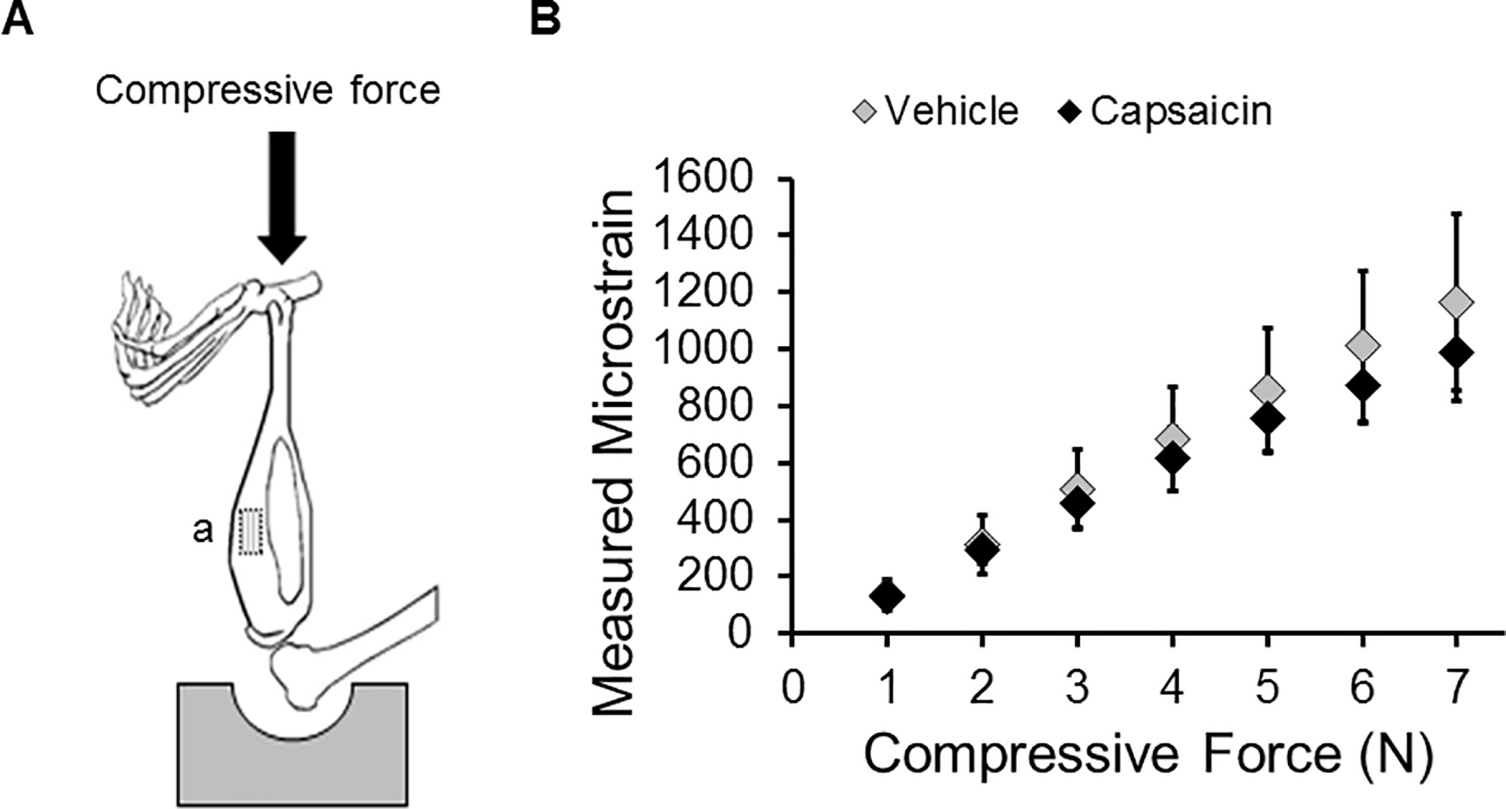
Maximum bone strain during tibial compression at the anteromedial tibial surface. (**A**) A diagrammatic representation shows the approximate location of the strain gauge (a). (**B**) Maximum bone strain measured during tibial compression at compressive magnitudes from 1–7 N. No significant differences in measured strain were observed between capsaicin- and vehicle-treated mice at any of the compressive forces tested.

Bone strain measured on the anteromedial surface of the tibia was not different for capsaicin- and vehicle-treated mice for compressive forces of 1–7 N (Fig 4B), though vehicle-treated mice exhibited a trend toward greater bone strain at equivalent compressive loads relative to capsaicin-treated mice. Power analysis revealed that for these means and standard deviations, 95 mice per group would be required to show significant differences at 3 N loading, while 31 mice per group would be required for 7 N (80% power, alpha = 0.05). Therefore, all mice were loaded to the same target force of 3 N or 7 N for the investigation of bone adaptation, regardless of treatment (capsaicin or vehicle).

### Hot-plate analgesia testing

**Fig 5:**
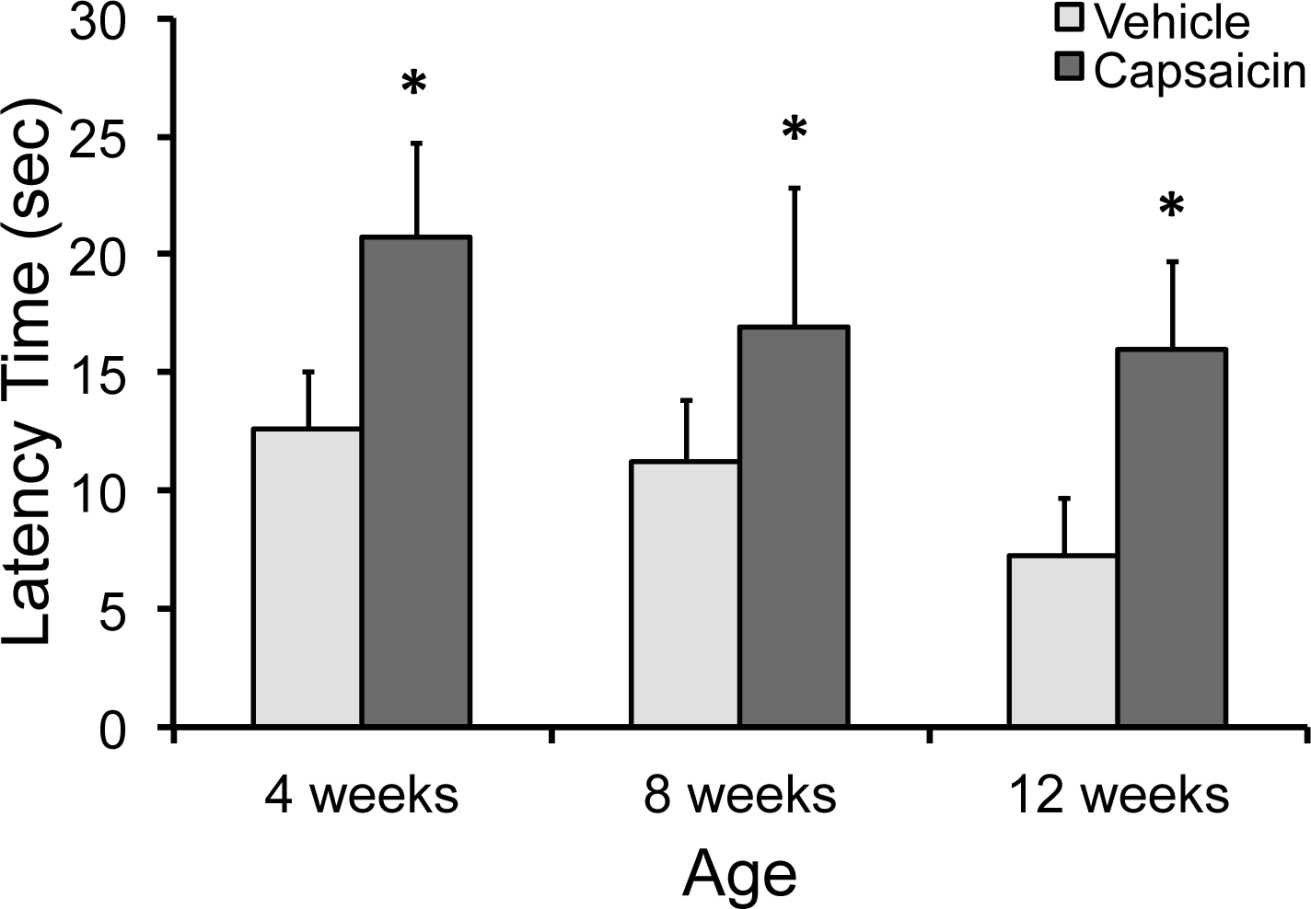
Hot-plate test of thermal analgesia. Hot-plate analgesia testing of vehicle- and capsaicin-treated mice was performed at 4, 8, and 12 weeks of age to verify decreased peripheral sensory nerve function prior to tibial compression. Capsaicin-treated mice exhibited significantly longer latency times than vehicle-treated mice at all time points when exposed to a constant 55°C thermal stimulus, indicating decreased thermal sensitivity. * indicates significant differences between capsaicin- and vehicle-treated mice (p < 0.05).

Latency time of capsaicin-treated mice was significantly longer than that of vehicle-treated mice at all ages tested, consistent with reduced peripheral sensory nerve function resulting from neonatal capsaicin treatment (Fig 5). At 12 weeks of age, latency time of capsaicin-treated mice was 120% longer than that of vehicle-treated mice.

### Micro-computed tomography analysis of bone structure

MicroCT analysis revealed that mechanical loading did not significantly affect trabecular bone volume fraction (BV/TV) of the tibial metaphysis, although the microstructure of trabecular bone was slightly altered. For example, loaded and contralateral tibias displayed a similar BV/TV at each compressive force (Table 1), however loaded tibias had greater trabecular thickness and reduced trabecular number relative to contralateral tibias.

**Table 1:** Trabecular bone parameters assessed at the tibial metaphysis using microCT.

Treatment Group	BV/TV		Tb.Th		Tb.N	
	Control	Loaded	Control	Loaded	Control	Loaded
***3N compressive force***						
**Vehicle**	0.107±0.013	0.110±0.011	0.0440±0.0016	0.0451±0.0022	4.28±0.244	4.25±0.164
**Capsaicin**	0.105±0.007	0.108±0.003	0.0421±0.0017	0.0422±0.0020	4.35±0.163	4.40±0.135
***7N compressive force***						
**Vehicle**	0.119±0.011	0.112±0.014	0.0449±0.0028	0.0503±0.0029	4.20±0.242	3.89±0.223
**Capsaicin**	0.127±0.008	0.129±0.017	0.0430±0.0014	0.0471±0.0031	4.51±0.280	4.30±0.351

Data reported as mean±SD.

**Fig 6:**
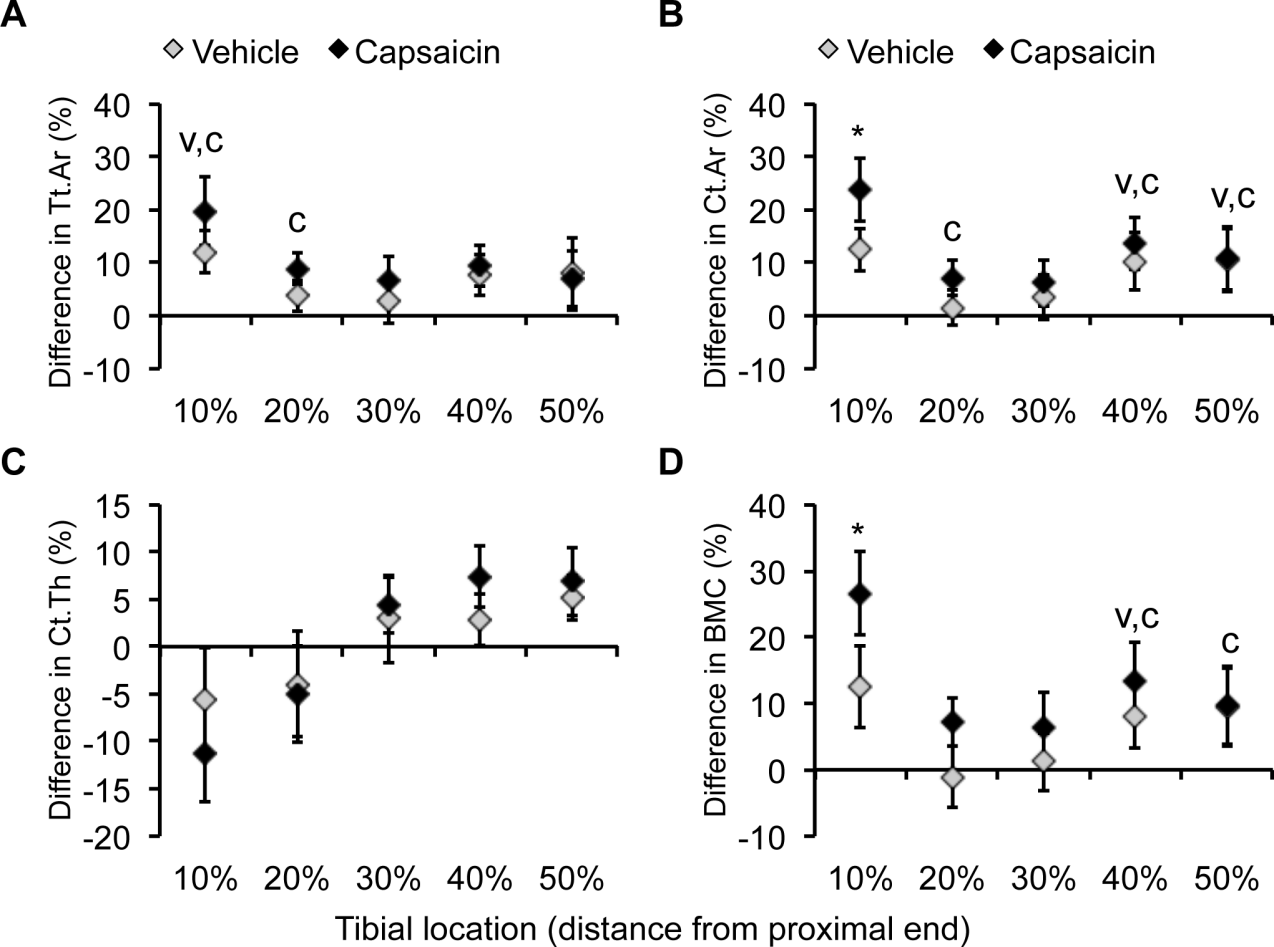
Micro-computed tomography analysis of cortical bone changes. Significant changes in cortical bone microstructure were identified at different locations along the length of the tibias after two weeks of 7 N tibial compression. Diamonds represent the average change of loaded compared to control tibias with error bars indicating standard deviation. (**A**) Total cross-sectional area (Tt.Ar) was significantly increased in loaded tibias at all locations except 30%. (**B**) Changes in cortical bone area (Ct.Ar) mirrored changes in Tt.Ar. (**C**) Cortical thickness (Ct.Th) decreased at more proximal locations along the tibia. (**D**) Bone mineral content (BMC) was significantly altered by tibial compression and varied by location on the tibia. **V**: loading caused a significant increase in vehicle-treated mice, p<0.05. **C**: loading caused a significant increase in capsaicin-treated mice, p<0.05. *: the response was significantly different between vehicle- and capsaicin-treated mice.

Tibial compression significantly affected cortical bone of loaded tibias, with adaptation dependent on both the compressive force and location along the tibia (Fig 6, Table 2). Compression at 7 N generated a larger cortical bone response than 3 N compression. For example, at 10% of the tibia length, Tt.Ar increased 16% at 7 N compared to 3.3% at 3 N. In addition, BMC increased only 1.5% in tibias loaded at 3 N, while the increase was six times higher in tibias loaded at 7N. Location along the tibia also significantly affected the adaptive response of cortical bone. The largest changes were observed at 10% of the tibia length, whereas at 30% insignificant changes were often observed. The increase in Tt.Ar at 10% was accompanied by a 14% increase in Me.Ar at the 7N force. At 20%, the increase in Me.Ar was reduced 8.2%. Changes in Ct.Th also varied by location, with loaded tibias becoming thinner at 10% and thicker at 50% of their length. Capsaicin- and vehicle-treated mice typically displayed similar trends in adaptation to increased loading. However, tibias of capsaicin-treated mice demonstrated greater changes at some locations. For example, at 20% of bone length, capsaicin-treated mice exhibited an 8.8% increase in Tt.Ar, while vehicle-treated mice showed no significant change. Furthermore, the increase in BMC at 10% was almost twofold higher for capsaicin-treated mice compared to vehicle-treated mice.

**Table 2:** Cortical bone parameters assessed along control and loaded tibias using microCT.

Tibial Location	Treatment Group	Total area^a^^,^^b^^,^^c^ (mm^2^)		Medullary area^a^ (mm^2^)		Bone mineral content^a^^,^^b^ (mg HA)	
		Control	Loaded	Control	Loaded	Control	Loaded
***3N compressive force***							
**10%**	**Vehicle**	2.82±0.18	2.97±0.28	1.91±0.17	2.02±0.24	0.60±0.03	0.63±0.04
	**Capsaicin**	2.65±0.15	2.67±0.16	1.79±0.13	1.80±0.11	0.60±0.04	0.57±0.05
**20%**	**Vehicle**	1.67±0.14	1.70±0.11	0.84±0.12	0.85±0.02	0.53±0.02	0.55±0.03
	**Capsaicin**	1.57±0.06	1.56±0.05	0.78±0.05	0.77±0.04	0.50±0.02	0.51±0.02
**30%**	**Vehicle**	1.40±0.09	1.39±0.06	0.66±0.06	0.65±0.05	0.49±0.03	0.49±0.02
	**Capsaicin**	1.31±0.04	1.30±0.05	0.61±0.02	0.61±0.03	0.46±0.02	0.46±0.02
**40%**	**Vehicle**	1.22±0.08	1.22±0.06	0.54±0.04	0.54±0.03	0.45±0.03	0.46±0.02
	**Capsaicin**	1.15±0.06	1.15±0.05	0.51±0.03	0.50±0.02	0.43±0.02	0.43±0.02
**50%**	**Vehicle**	0.94±0.05	0.92±0.06	0.37±0.03	0.36±0.04	0.40±0.02	0.39±0.02
	**Capsaicin**	0.89±0.06	0.87±0.04	0.35±0.03	0.34±0.03	0.37±0.02	0.37±0.02
***7N compressive force***							
**10%**	**Vehicle**	3.04±0.10	3.41±0.14*	2.03±0.09	2.27±0.12	0.65±0.03	0.73±0.04*
	**Capsaicin**	2.79±0.18	3.33±0.12*	1.85±0.13	2.17±0.10	0.60±0.04	0.75±0.04**
**20%**	**Vehicle**	1.81±0.07	1.87±0.08	0.90±0.07	0.96±0.06	0.58±0.03	0.57±0.02
	**Capsaicin**	1.67±0.06	1.82±0.06*	0.81±0.04	0.90±0.06	0.54±0.03	0.58±0.02
**30%**	**Vehicle**	1.52±0.04	1.57±0.06	0.72±0.04	0.74±0.04	0.53±0.02	0.54±0.02
	**Capsaicin**	1.42±0.09	1.51±0.05	0.64±0.05	0.68±0.04	0.51±0.03	0.54±0.01
**40%**	**Vehicle**	1.30±0.04	1.40±0.05	0.58±0.03	0.61±0.03	0.49±0.02	0.53±0.03*
	**Capsaicin**	1.22±0.06	1.33±0.06	0.52±0.03	0.54±0.03	0.47±0.03	0.53±0.03*
**50%**	**Vehicle**	1.00±0.03	1.08±0.06	0.39±0.02	0.41±0.03	0.43±0.01	0.47±0.03*
	**Capsaicin**	0.95±0.06	1.01±0.05	0.36±0.03	0.37±0.03	0.41±0.02	0.45±0.02

Data reported as mean±SD

^a^: significant leg*VOI interaction, 7N compressive force

^b^: significant treatment*leg*VOI interaction, 7N compressive force

^c^: significant leg*VOI interaction, 3N compressive force

*: significant difference between control and loaded, p<0.05

**:significant difference between capsaicin and vehicle response, p<0.05

### Dynamic histomorphometry of bone formation

**Fig 7:**
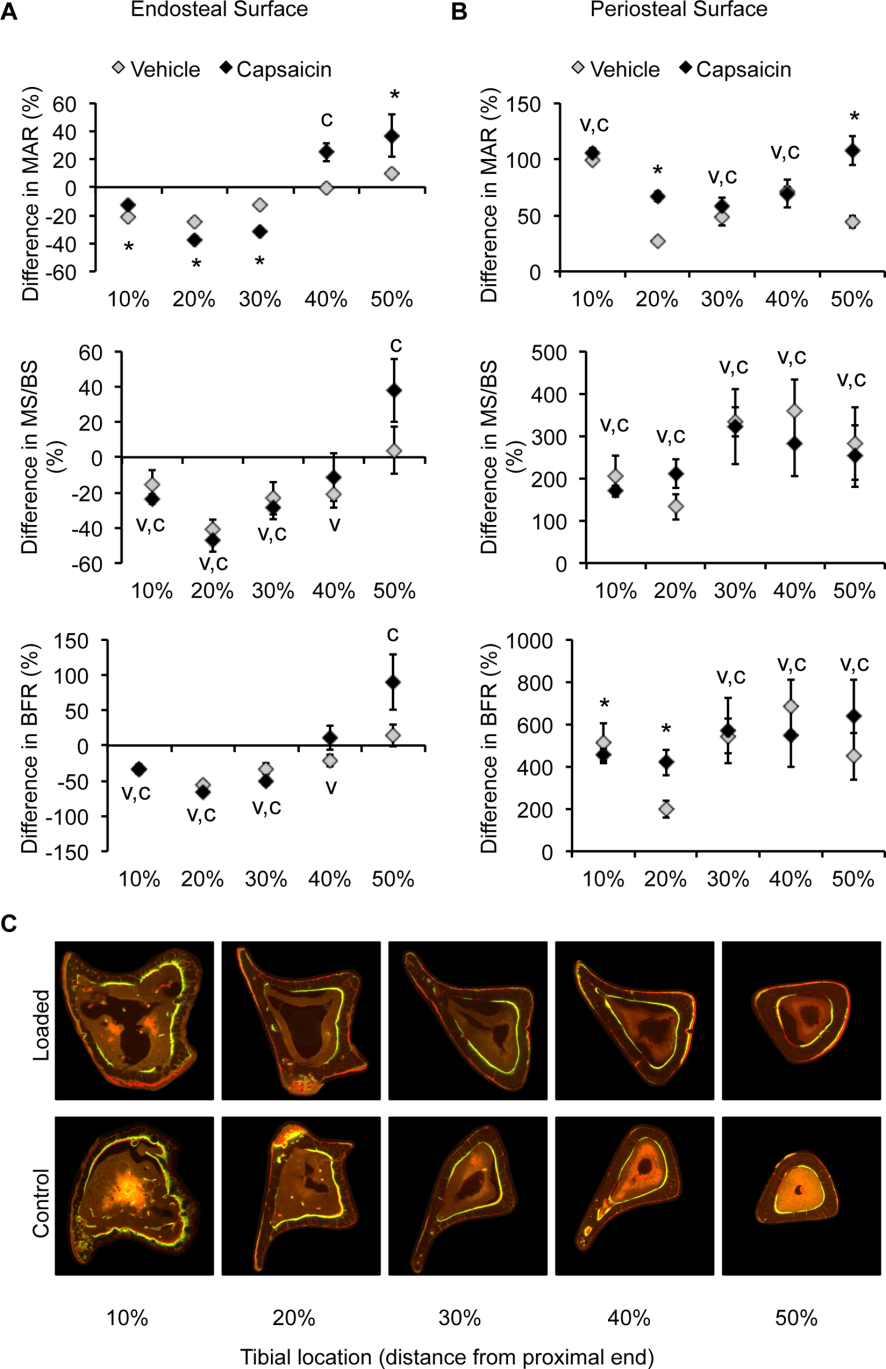
Dynamic histomorphometric analysis of bone formation in capsaicin- and vehicle-treated mice. Mineral apposition rate (MAR), percent mineralizing surface (MS/BS), and bone formation rate (BFR/BS) were quantified for the endosteal (**A**) and periosteal surfaces (**B**) in mice loaded with tibial compression at 7 N magnitude. Fluorescent images (**C**) show cortical bone from the control and loaded tibias of a vehicle-treated mouse. **V**: loading caused a significant difference in tibias of vehicle-treated mice, p<0.05. **C**: loading caused a significant difference in tibias of capsaicin-treated mice, p<0.05. *: the response was significantly different between vehicle- and capsaicin-treated mice.

Dynamic histomorphometric analysis revealed that 7 N tibial compression altered bone formation parameters on the endosteal and periosteal surfaces of loaded tibias (Table 3). In general, bone formation increased on the periosteal surface of the tibias and decreased on the endosteal surface at locations closest to the proximal metaphysis. There was a significant effect of treatment on the response of MAR to increased loading, with capsaicin-treated mice displaying a larger response compared to vehicle-treated mice at many tibial locations (Fig 7). For example, at 20% of the tibia length, the loaded tibias of capsaicin-treated mice had an average decrease in MAR of 37% compared to a 24% decrease in MAR in vehicle-treated mice. While MAR was lower in loaded tibias compared to controls at 10, 20 and 30% of the tibia length on the endosteal surface, MAR was increased at all measured locations on the periosteal surface. At 20%, capsaicin-treated mice again demonstrated a larger response to increased loading with an approximately 2.5 times larger increase in MAR than vehicle-treated mice on the periosteal surface. There was a significant effect of loading on MS/BS that varied with location along the tibia. However, treatment was not a factor in the response of MS/BS on the periosteal surface. Loading also caused a significant increase in BFR/BS at all locations on the periosteal surface, with a significantly greater response in capsaicin-treated mice at 20% of the tibia length. Tibial compression at 3 N caused a significant decrease in MAR on the endosteal surface at 10 and 20%, but the effect did not differ between treatment groups. Furthermore, the lower magnitude compression did not significantly affect MS/BS or BFR/BS on either surface of the loaded tibias.

**Table 3:** Bone formation parameters assessed using dynamic histomorphometry.

**Tibial Location**	**Treatment Group**	**MAR**^**a**^^**,**^^**b**^ **(μm/day)**		**MS/BS**^**a**^^**,**^^**b**^ **(%)**		**BFR**^**a**^^**,**^^**b**^ **(μm**^**3**^**/μm**^**2**^**/day)**	
		**Control**	**Loaded**	**Control**	**Loaded**	**Control**	**Loaded**
**Endosteal Surface** ***7N compression***							
**10%**	**Vehicle**	2.05±0.03	1.62±0.01*	62±1	53±5*	1.28±0.03	0.85±0.08*
	**Capsaicin**	1.96±0.04	1.71±0.02**	65±2	50±3*	1.27±0.05	0.85±0.05*
**20%**	**Vehicle**	1.30±0.01	0.98±0.03*	51±3	30±3*	0.66±0.03	0.30±0.03*
	**Capsaicin**	1.59±0.05	0.99±0.03**	49±5	26±3*	0.77±0.07	0.25±0.03*
**30%**	**Vehicle**	1.11±0.01	0.97±0.02*	53±8	40±2*	0.59±0.09	0.39±0.03*
	**Capsaicin**	1.25±0.02	0.85±0.03**	51±4	36±2*	0.64±0.05	0.31±0.03*
**40%**	**Vehicle**	1.07±0.02	1.07±0.02	49±6	39±1*	0.53±0.07	0.41±0.02*
	**Capsaicin**	0.79±0.03	0.98±0.03*	38±2	33±4	0.30±0.02	0.33±0.04
**50%**	**Vehicle**	1.01±0.03	1.11±0.02*	36±6	37±4	0.37±0.06	0.41±0.04
	**Capsaicin**	0.66±0.02	0.90±0.09**	26±2	37±6*	0.17±0.01	0.33±0.08*
		**MAR**^**a**^^**,**^^**b**^ **(μm/day)**		**MS/BS**^**a**^ **(%)**		**BFR**^**a**^^**,**^^**b**^ **(μm**^**3**^**/μm**^**2**^**/day)**	
		**Control**	**Loaded**	**Control**	**Loaded**	**Control**	**Loaded**
**Periosteal Surface** ***7N compression***							
**10%**	**Vehicle**	0.91±0.01	1.82±0.02*	10±1	29±3*	0.09±0.01	0.52±0.05*
	**Capsaicin**	1.02±0.02	2.09±0.03*	12±0	33±2*	0.13±0.01	0.70±0.05**
**20%**	**Vehicle**	1.53±0.02	1.95±0.02*	19±2	43±4*	0.29±0.03	0.85±0.08*
	**Capsaicin**	1.06±0.02	1.77±0.02**	15±1	47±3*	0.16±0.01	0.82±0.05**
**30%**	**Vehicle**	0.75±0.03	1.11±0.02*	10±1	41±2*	0.07±0.01	0.46±0.02*
	**Capsaicin**	0.82±0.03	1.29±0.03*	10±1	44±7*	0.09±0.01	0.56±0.09*
**40%**	**Vehicle**	0.87±0.02	1.49±0.02*	11±1	52±5*	0.10±0.01	0.77±0.08*
	**Capsaicin**	0.82±0.04	1.39±0.10*	14±1	53±8*	0.11±0.01	0.74±0.14*
**50%**	**Vehicle**	0.77±0.02	1.11±0.04*	14±4	52±5*	0.11±0.03	0.58±0.07*
	**Capsaicin**	0.55±0.03	1.14±0.03**	17±1	60±11*	0.09±0.01	0.69±0.13*

Data reported as mean±SD

^a^: significant leg*VOI interaction, 7N compressive force

^b^: significant treatment*leg*VOI interaction, 7N compressive force

*: significant difference between control and loaded, p<0.05

**:significant difference between capsaicin and vehicle response, p<0.05

### ELISA analysis of neuropeptide concentrations in bone

**Fig 8:**
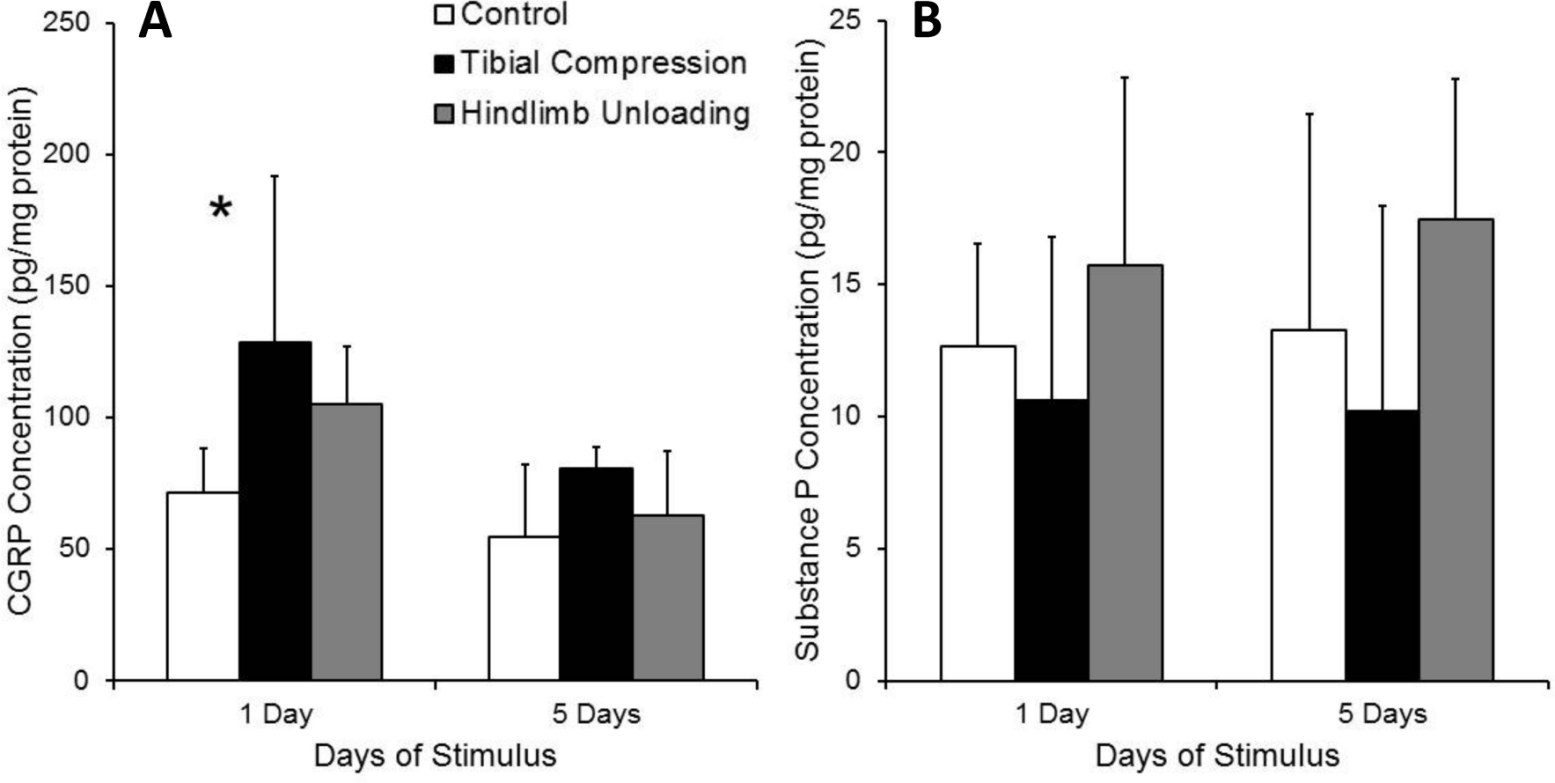
Neuropeptide concentrations in bones of mice subjected to tibial compression or hindlimb unloading. (**A**) CGRP concentration in the bones of mice subjected to tibial compression or hindlimb unloading. Main effects of time point and type of stimulus (tibial compression or hindlimb unloading) were observed, and a significant increase in CGRP concentration was observed following 1 day of tibial compression. (**B**) SP concentration in the bones of mice subjected to tibial compression or hindlimb unloading. A significant main effect type of stimulus was observed, but no significant differences were observed between any experimental groups.

Concentration of CGRP in bone was somewhat altered by increased and decreased mechanical loading (Fig 8A). The concentration of CGRP in the tibias was significantly higher after 1 day of tibial compression or hindlimb unloading than after 5 days (main effect of time point; p = 0.0005). The type of loading stimulus (tibial compression or hindlimb unloading) also significantly affected CGRP concentration (main effect of stimulus type; p = 0.0038). After one day of tibial compression, average CGRP concentration was increased 66% relative to control tibias (128 pg/mg protein vs. 71.5 pg/mg protein in controls; p = 0.0193). However, CGRP concentration did not differ significantly from control tibias after 5 days of tibial compression, and CGRP in hindlimb unloaded limbs was not significantly different from controls at either time point.

SP concentrations were less affected by altered mechanical loading than those for CGRP (Fig 8B). The type of stimulus (tibial compression or hindlimb unloading) had a significant effect on SP bone concentration (main effect of stimulus type; p = 0.047), with hindlimb unloaded tibias exhibiting 37% higher SP concentration than tibial compression tibias. The duration of tibial compression or hindlimb unloading did not significantly affect SP concentration, and no significant differences were observed between experimental groups using post hoc analysis.

## Discussion

The purpose of this study was to investigate the role of sensory nerves in adaptation of bone to increased mechanical loading. We found that tibial compression, particularly at 7 N magnitude, caused an increase in cortical bone area in the loaded tibia, accompanied by changes in bone formation parameters. This adaptive response was dependent on location along the tibia, and the magnitude of this response was generally similar, or even somewhat greater in capsaicin-treated mice than in vehicle-treated mice. This possible increased response in capsaicin-treated mice conflicts with our initial hypothesis that reduced sensory nerve function would impair the adaptation of bone to increased loading. We also investigated concentrations of CGRP and SP in bone in response to tibial compression or hindlimb unloading, and found that tibial compression resulted in a significant increase in CGRP concentration after one day of loading. Altogether these findings suggest that sensory nerves and neuropeptides may play a direct role in bone adaptation to mechanical stimuli.

The study of bone adaptation to loading was performed at two different compressive loads. Two weeks of 3 N tibial compression generated no significant adaptation response in cortical bone assessed with μCT, and no significant changes in bone formation measured by dynamic histomorphometry. Similarly, Fritton et al. observed a 1.4% increase in whole bone mineralized tissue volume when using this compressive force, which increased to 3.4% when the loading was conducted for an additional 4 weeks [30]. Although these researchers were able to detect an increase in BMC at 10% of the tibia length using 3 N loading, we were only able to observe changes with the 7 N compressive force. Interestingly, we did not observe significant changes in trabecular bone that others have reported in studies of bone adaptation [30, 31, 40]. The small magnitude of changes in bone structure may be a result of differences in tibial compression systems. For example, we have found that higher compressive forces using our configuration may lead to knee injury and subsequent trabecular bone loss [32]. A group using a similar configuration observed a 20% loss in BV/TV at the proximal metaphysis in tibias loaded to 8 N [40]. Another group using a different configuration for tibial compression found an increase of 44.5% in BV/TV in tibias loaded to a target compressive force of 13.5 N [38].

Location on the tibia influenced the bone adaptive response, consistent with the morphology of the mouse tibia resulting in different strain patterns along its length [35, 37]. We observed the largest changes in Tt.Ar and BMC at 10% of the tibia length, where others have reported the largest compressive magnitude [37]. The shift we observed in bone formation parameters at 30% could be a result of a shift in strain from compression to tension. Tibia surface also factored into the adaptation results, with the endosteal surface of loaded tibias displaying a decrease in BFR/BS at 10–30% compared to control tibias. This is consistent with the observation of increased Tt.Ar and Me.Ar from μCT. Tibial compression caused outward expansion of the cortex, with increased bone formation on the periosteal surface and decreased bone formation on the endosteal surface, which may help the tibia adapt to the demands of greater compression [41]. The changes we observed in cortical bone assessed with μCT are consistent with changes in bone formation parameters assessed with dynamic histomorphometry. The increase in Tt.Ar and BMC at 10% of the tibia length were attended by a large increase in MAR and BFR/BS on the periosteal surface. A similar trend occurred at 50% of the tibia length. The increase in BFR/BS on the periosteal surface and decrease on the endosteal surface could account for the modest increase in Tt.Ar at 10% of the tibia length.

Contrary to our initial hypothesis, capsaicin treatment caused similar or greater changes in parameters such as Tt.Ar, BMC and MAR in response to increased loading. A possible explanation for this observation is that neuropeptide concentrations are antagonistic to the actions of bone cells during adaptation *in vivo*. In a study of ulnar compression in rats, researchers found that increases in bone area were accompanied by decreases in bone concentrations of CGRP [18]. As CGRP has an anabolic effect on osteoblasts, the reduction from baseline in response to loading appears to work against the bone forming action of osteoblasts. Similarly, another study found that hindlimb casting in rats caused elevated CGRP concentrations in the sciatic nerve [19]. The bone loss associated with immobilization may be antagonized by increased concentrations of this neuropeptide. By reducing nerve function with capsaicin treatment, we may be limiting the antagonistic role of neuropeptides in bone adaptation, allowing a greater response to loading.

This study used the same compressive forces for both vehicle-treated and capsaicin-treated mice. Ideally, the magnitude of compressive force would be normalized so that the same magnitude of bone strain is induced in the two groups. Capsaicin-treated mice have slightly smaller bones, with 4–11% smaller average Tt.Ar, Me.Ar, and Ct.Ar in the non-loaded limb (Table 2); therefore one would expect that compressive loading would induce greater bone strains in these mice than in vehicle-treated mice. However, our study of tibial bone strain during compressive loading showed that vehicle-treated mice exhibited *greater* bone strain than capsaicin-treated mice at all compressive magnitudes (Fig 4). Therefore it was not possible for us to adjust the compressive loading magnitude based on this data. It is unclear why we observed this pattern of bone strain in capsaicin- and vehicle-treated mice. It is possible that capsaicin-treated mice had stiffer bone tissue than vehicle-treated mice. However, this is unlikely based on our previous study [26], which showed few differences in the mechanical properties of bones from capsaicin- and vehicle-treated mice. It is also possible that neonatal capsaicin treatment resulted in shape changes to the bone that resulted in lower strains being recorded at the gage site. A more inclusive (i.e., whole bone) method of strain measurement [42] may be needed to detect potential differences in the bone strain patterns between the two groups. Performing the strain gage study with additional mice may also have allowed us to detect more consistent differences in bone strain, however our animal numbers for this portion of the study were consistent with previous studies that conducted similar analyses [30, 31, 40], and power analysis of this data indicated that >30 mice per group would be needed to observe significant differences between capsaicin- and vehicle-treated mice. Alternatively, we could have normalized compressive strain to average tibial cross-sectional area, body weight, or other morphological parameters. Normalizing the compressive forces to these parameters may have affected the results of this study.

CGRP and SP are two of the main neuropeptides found in the sensory nerves innervating bone. CGRP is a 37 amino acid peptide produced from alternative splicing of the calcitonin gene. Nerves containing CGRP appear in proximity to bone lining and precursor cells in the periosteum. CGRP containing nerves are also found near the epiphyseal plate, in bone marrow, and around blood vessels in Volkmann’s canals. Near epiphyseal trabecular bone, CGRP fibers terminate in free endings near osteoblasts and osteoclasts, which both express receptors for the neuropeptide [43]. Osteoblasts respond to CGRP with an increase in intracellular cAMP and corresponding changes in cell morphology and function [7]. CGRP also affects osteoclasts, inhibiting their function and the recruitment of macrophages into osteoclast-like cells. *In vivo*, administration of high doses of CGRP inhibits bone resorption and lowers serum concentrations of calcium [44]. CGRP neurons in bone are more numerous than SP containing neurons, although the 11 amino acid peptide also potentially plays an important role in bone. The traditional role of SP is transmission of pain signals in nociceptive afferents. Nerves containing SP are found in the periosteum of long bones and in the bone marrow next to blood vessels, where they separate and terminate in free endings. Osteoblasts express the SP receptor neurokinin-1 and exposure to SP increases osteoblast function in vitro [15]. Osteoclasts also have receptors for SP, and exposure *in vitro* stimulates calcium influx and bone resorption [16]. Both CGRP and SP often appear together in the small sensory neurons, including Aδ and C fibers. CGRP may facilitate SP release and enhance activation [17].

Our finding of increased CGRP concentration in bone following one day of tibial compression is in contrast with a previous study that showed a decrease in bone CGRP in response to cyclic compression [18]. The reason for these differing observations is unclear. Our study used tibial compression in mice vs. ulnar compression in rats in the study by Sample et al. However, both studies found a decrease in SP following mechanical loading, though the differences observed in our study were not statistically significant. A study by Guo et al. found that both CGRP and SP levels were increased in the sciatic nerve after 4 weeks of decreased mechanical loading (cast immobilization) [19]. Our findings are generally in agreement with this study, though the increases in CGRP and SP observed with hindlimb unloading in our mice were not statistically significant.

The relatively small number animals in this study presents a limitation for the interpretation of our findings. Especially with respect to SP, bone concentrations of these neuropeptides were small. Detection of a minor change in concentration resulting from the different mechanical stimuli may require the use of much higher animal numbers to achieve statistical significance. For example, detecting a significant 15% difference in substance P concentration between control tibias and 1 day compressed tibias would require 162 mice. Another limitation is that while we measured bone concentration, we do not know the origin of these neuropeptides. Osteoblasts themselves express CGRP and might alter their expression as an adaptive mechanism to the new loading environment. Such action would suggest an autocrine or paracrine response rather than a neural one. A final limitation is that we did not have a separate group of mice subjected to normal cage activity to serve as a universal control. Instead we used the left tibias of mice subjected to tibial compression. This is a potentially important limitation if neuropeptides are released from the loaded tibia and diffuse into the circulation to act as systemic factors.

Our investigation of the role of sensory nerves on bone adaptation is limited by the fact that we did not investigate gait, muscle function, or activity level in capsaicin- and vehicle-treated mice. There were no obvious changes in gait or activity level in capsaicin-treated mice, but these were not directly quantified. A previous study investigating the effects of capsaicin treatment in adult rats measured gait, activity level, and muscle function following treatment [25]. They found no significant differences between capsaicin- and vehicle-treated rats in spontaneous locomotor activity over a 24-h period, maximum hindpaw weight bearing, or mean calf muscle mass (gastrocnemius and soleus). Our study also did not directly quantify the effect of capsaicin treatment on nerve function or neuropeptide concentrations in bone, nor did we investigate changes to the structure or distribution of sensory nerves in bone following capsaicin treatment, or following mechanical loading or disuse. Instead, we measured a functional outcome, the response of treated mice to a thermal stimulus. Others have evaluated the chemosensitivity of the cornea to assess sensory denervation resulting from neonatal capsaicin treatment [23, 24]. A study of capsaicin treatment in adult rats showed that unmyelinated sensory axons were destroyed and neuropeptide concentrations reduced in the tibia [25]. The effect of sensory nerves on bone may be limited to a local interaction, with neuropeptide concentrations affecting bone metabolism. While capsaicin-treated mice demonstrated reduced thermal sensitivity at the periphery, they may have developed a compensatory mechanism in bone. Neonatal capsaicin treatment may have limited functionality for studies of bone metabolism or adaptation, since there is incomplete ablation of sensory nerves with notable mouse-to-mouse variability. Genetic mouse models targeting specific factors (e.g., TRPV1, NF1, SP) may be more effective at consistently modulating the function of sensory nerves.

## Conclusions

Contrary to our initial hypothesis, reduced peripheral sensory nerve function did not hinder the adaptation of bone to increased mechanical loading in this study. Capsaicin-treated mice were able to mount an adaptive response that was generally similar or even somewhat greater than that of vehicle-treated mice. It is possible that sensory nerves do not play a direct role in the bone adaptation response *in vivo*. Alternatively, it is possible that sensory nerves exert a modulating effect on bone in response to mechanical stimuli, with altered neuropeptide concentration acting to moderate bone formation in cases of increased loading. Our investigation of neuropeptide concentrations in bone revealed that altered mechanical environments have the capacity to alter neuropeptides expression. Traditionally classified as transmitters of sensory stimuli, neuropeptides may have an additional role in bone, where they have the potential to regulate the bone cell response necessary for adaptation.

## Supporting information

S1 File Mouse body weight data. (XLSX)

S2 File Antero-medial tibial surface bone strain results. (XLSX)

S3 File Hot plate analgesia test results. (XLSX)

S4 File Micro-computed tomography results for mice loaded at 3N compression. (XLS)

S5 File Micro-computed tomography results for mice loaded at 7N compression. (XLS)

S6 File Cortical bone dynamic histomorphometry data. (XLSX)

S7 File Bone neuropeptide concentration ELISA results. (XLSX)
